# 

**Published:** 1783

**Authors:** 


### THE LONDON MEDICAL JOURNAL.

### VOLUME THE FOURTH.

Surgeon General's Office
LIBRARY.
[illegible]
Washington, D.C.

LONDON:

Printed for the EDITOR by W. RICHARDSON,

AND SOLD

By JOSEPH JOHNSON, N° 72, St. Paul's Church Yard.

M DCC LXXXIV.

### PROMOTED.

Lately, His Grace the Duke of Montagu, Sir Joseph Banks, Bart. P. R. S. and Dr. Peter Camper to be honorary fellows of the Royal College of Phyficians at Edinburgh.—Dr. Lallemand to be Prefident of the Royal College of Phyficians at Nancy in the room of the late Dr. Harmant.—Dr. Baldinger, profeffor of phyfic at Gottingen, to be firft phyfician to the Landgrave of Heffe Caffel.—Dr. John Ehrmann to be profeffor of phyfic at Strafburgh in the room of the late Dr. Pfeffinger.

1782, *Sept.* 12. Mr. John Forde to be furgeon to the Royal Leinfter provincial regiment of foot.—13. Mr. E. L. Ledgwick to be furgeon to the Ulfter provincial regiment of foot.

*Dec.* 28. Mr. Thomas Venour, furgeon of the 75th, to be furgeon of the 18th regiment of foot, in the room of Mr. Richard Babington. —Mr. Patrick Drummond, hofpital mate, to be furgeon of the 52d regiment of foot in the room of Mr. Patrick Dundon.—Mr. Everard Home, hofpital mate, to be furgeon to the 1ft battalion of the 60th regiment of foot in the room of Mr. Peter Walfh.—Mr. Edward M'Allifter lifter to be furgeon of the 75th regiment of foot in the room of Mr. Thomas Venour.

1783, *Jan.* 9. Mr. William Thompfon, furgeon of the Victory, to be furgeon to the Dock-yard at Deptford in the room of Mr. W. Troward, who is fuperannuated.—11. Mr. Gawin Hamilton to be furgeon of the 2d regiment of Dragoon guards.——14. Mr. Brifcoe, furgeon's mate, to be furgeon of the 100th regiment of foot.—Mr. William Cooper to be furgeon to Guy's Hofpital in the room of Mr. James Frank deceafed.——18. Mr. Thomas Richard Spence to be dentift in ordinary to his Majefty in the room of his father deceafed.—24. Mr. George Chandler to be furgeon to St. Thomas's Hofpital in the room of Mr. Waring deceafed.

*Feb.* 24. Mr. Henry Cline to be furgeon to St. Luke's Hofpital in the room of Mr. George Chandler.

*March* 21. Mr. Patrick Dillon to be furgeon to the 105th regiment of foot.

---

### D I E D.

Lately at Montpellier, M. Montet, an ingenious apothecary and member of the Royal

Society

Society of that city. This Inſtitution, by its charter, is incorporated with the Royal Academy of Sciences at Paris, and in conſequence of this union a paper is ſent every year by the Montpellier Society to be inſerted in the Memoirs of the Academy. For ſeveral years paſt this paper has been furniſhed by M. Montet, and by his will he has bequeathed the ſum of 6000 livres to the Society, the intereſt of which is to be paid annually to the member whoſe paper ſhall be deemed worthy of the ſame diſtinction.

1782, *Sept.* 23. Loſt in the Centaur Man of War, of which he was ſurgeon, Mr. Joſeph Williamſon.——29. Of a dyſentery, aged 59 years, Dominic Benedict Harmant, M. D. preſident of the Royal College of Phyſicians at Nancy, and author of an ingenious treatiſe on the noxious effects of burning charcoal.

*Oct.* At Aberdeen, aged 65 years, Sir Alexander Gordon, baronet, profeſſor of phyſic in King's College, and author of a Theſis *de variolis*, printed at Edinburgh in 1754.

*Dec.* 9. At Plymouth, Dr. Robert Perronneau, a native of South Carolina, and author of an ingenious Inaugural Theſis *de Menſtruorum Profluvio immodico*, printed at Edinburgh in

1775.

1775.—25. Mr. Richard Board, apothecary in Gracechurch-ſtreet, London.——30. In Fenchurch-ſtreet, Mr. James Frank, ſurgeon to Guy's Hoſpital, and one of the Court of Aſſiſtants of the Corporation of Surgeons, London. —31. At Colcheſter, Dr. Charles Hanning.

1783, *Jan.* 1. Mr. Pugh, ſurgeon in Southwark.—2. At his ſon's houſe in London, Mr. William Johnſton, formerly a ſurgeon and apothecary at Campden in Glouceſterſhire.——At Windſor, Mr. George Aylett, ſurgeon.—3. In Ranelagh-row, Pimlico, Mr. Joſeph Webb, ſurgeon. Ill health obliged him to retire from buſineſs ſeveral years before his death. He was the youngeſt ſon of the late Mr. Joſeph Webb, who for thirty years was ſurgeon to St. Bartholomew's Hoſpital.—6. Mr. Tryce, formerly an apothecary in Whitechapel.—7. In Great Tower ſtreet, Mr. John Waring, ſurgeon to St. Thomas's Hoſpital.——16. In Soho-ſquare, Mr. James Spence, ſenior, dentiſt in ordinary to his Majeſty.

*Feb.* 3. At Coventry, Mr. John Biby Hanker, ſurgeon and man-midwife.——5. Dr. Thomas Young, fellow of the R. C. of Phyſicians, and profeſſor of midwifery in the Univerſity of Edinburgh.—11. At Dunſe in Scotland, Mr. John Hall,

Hall, surgeon and apothecary.—23. In Tottenham-court Road, London, Mr. John Sheldon, senior, surgeon and apothecary.—24. At Deptford, Mr. William Troward, late surgeon of his Majesty's Dock-yard at that place.

*March* 8. At Brompton, near Chatham in Kent, suddenly, Mr. Gregory, surgeon and apothecary.

---

### SECTION IV.

### QUARTERLY CATALOGUE.

1. AN Historical account of two species of Lyncoperdon. By *Charles Bryant.* 8vo. *Wilkie*, London, 1782.

2. Oeconomiæ Animalis Dissertatio in usum Juventutis Medicæ. Auctore *Joanne Aikin*, in Academia Warringtoniensi Anatomiæ et Chemiæ Professore. 12mo. *Johnson*, London, 1782. 44 pages.

3. Observations on the means of preserving and restoring health in the West Indies. By *John Rollo.* 8vo. *Dilly*, London. 1782. 2s.

4. Candid animadversions on Dr. Lee's Narrative of a singular Gouty case. To which are prefixed strictures on Royal Medical Colleges; likelikewise a summary opinion of the late disorder called the influenza. By *W. Stevenson*, M.D. 8vo. *Dilly*, London, 1782. 6d.

5. A short history of the Brown-tail Moth, with a copper-plate, coloured from nature, representing the insect in its various states. By *William Curtis*, author of the Flora Londinensis. 4to. *White*, London, 1782. 1s. 6d.

6. Chemical Essays, vol. iii. By *Richard Watson*, D. D. F. R. S. 8vo. *Dodsley*, London, 1782. 4s.

7. A Brief history of the late expedition against Fort St. Juan, so far as it relates to the diseases of the troops: together with some observations on climate, infection, and contagion; and several of the endemial complaints of the West Indies. By *Tho. Dancer*, M. D. 4to. *Murray*, London, 1782. 2s. 6d.

8. Rapport fait par ordre du Gouvernement, sur un Memoire concernant la methode employée par feu *M. Doulcet*, Docteur Regent de la Faculté de Medecine de Paris, l'un des medecins de l'Hotel Dieu; dans le traitement d'une maladie qui attaque les femmes en couche, et que l'on connoit sous le nom de Fievre puerperale. Lû dans la seance de la Societé Royale de Medecine, tenue au Louvre le 6 Septembre, 1782. 4to. Paris, 1782. 8 pages.

 Our

Our readers will find a very judicious and ſatisfactory account of this report, and of the eſſay it relates to, by our ingenious correſpondent, Dr. Fothergill, in a former number of our Journal (vol. iii. p. 411).

9. A Report, made by order of Government, of a Memoir, containing a new, eaſy, and ſucceſsful method of treating the Child-bed or Puerperal fever, made uſe of by the late *M. Doulcet*, Doctor Regent of the Faculty at Paris, and one of the phyſicians of the Hotel-Dieu, read at a meeting of the Royal Medical Society, held at the Louvre, the 6th of September 1782. Tranſlated from the French. To which are added notes, containing a view of the nature and cauſes of this alarming and fatal diſeaſe. By *John Whitehead*, M. D. Member of the Royal College of Phyſicians, London, and Phyſician to the London Diſpenſary. Large 8vo. *Dilly*, London, 1783. 40 pages. 1s.

We have here an accurate tranſlation of the preceding article, with a preface by the tranſlator, and a conſiderable number of uſeful and intereſting notes. Another Engliſh tranſlation of the ſame piece (without notes) has been publiſhed by Dr. N. Maillard, in 42 pages, 8vo. price 1s. 6d.

10.

10. Obſervationum botanicarum ſpecimen. Auctore *G. W. F. Panzero*, M. D. 8vo. Nuremberg, 1782. 56 pages.

11. Traitement de la maladie miliaire epidemique et contagieuſe qui regne a Caſtelnaudary; *i. e.* Treatment of the epidemic and contagious miliary fever that prevails at Caſtelnaudary. 8vo. Toulouſe, 1782. 8 pages.

12. Memoire de meſſieurs les medecins de Caſtelnaudary, Souze, Carcaſſonne, and Montreal, concernant la maladie qui regne actuellement à Caſtelnaudary: *i. e.* Memorial of the phyſicians of Caſtelnaudary, &c. concerning the diſeaſe now prevalent at Caſtelnaudary. 4to. Toulouſe, 1782. 8 pages.

13. Methode pour le traitement de la fievre eruptive miliaire ou ſuette miliaire: *i. e.* Method of treating the eruptive or ſweating miliary fever. By M. *Gallet Dupleſſis*, phyſician at Carcaſſonne. 8vo. Toulouſe, 1782.

14. Traitement de la fievre miliaire epidemique à Toulouſe ſur la fin de Mai 1782; *i. e.* Treatment of the miliary fever which prevailed epidemically at Toulouſe towards the end of May 1782. 8vo. Toulouſe, 1782. 8 pages.

15. Reflexions sur la nature et le traitement de la maladie qui regne dans le Haut Languedoc. *i. e.* Reflexions on the nature and treatment of the diſorder that prevails in Upper Languedoc. Read at the Royal Medical Society, June 4, 1782. 4to. Paris, 1782.

16. De Reſina Elaſtica Cajennenſi. Auctore *A. Juliano.* 8vo. Utrecht, 1781. 72 pages.

We have here an account of a great number of experiments made with this reſinous ſubſtance. A piece of it, two inches long and weighing only fourteen grains, ſupported a weight of nine pounds and three quarters. The piece ſtretched to the length of eight inches and did not break, till another quarter of a pound was added. Spirit of wine diſſolved only a very ſmall quantity of it, but very concentrated oil of vitriol ſeemed to diſſolve it intirely, as likewiſe did vitriolic æther. The thin oil that it yields by diſtillation inflamed with ſmoaking ſpirit of nitre. When a ſolution of it in this acid was ſuperſaturated with alkali, the ſolution became of a bright red colour and was eaſily diluted with water. A long continued heat was required to diſſolve it in linſeed, almond, or olive oil; and a long boiling when any of the eſſential oils was employed. Caſtor oil did not ſeem to act upon it. Among other menmenſtrua for this ſubſtance the author ſpeaks of a new balſam produced at Surinam by a tree called *koupa*.

17. Raccolta di opuſculi Fiſico-medici. *i. e.* A collection of Phyſico-medical eſſays, vol. 22d. Florence. 12mo. 1780. 358 pages.

This work, the editor of which is Dr. Targioni, is now reſumed again, after an interruption of two years. Among other papers in the preſent volume are the following: 1. An eſſay to prove that Phthiſis is not a contagious diſeaſe, by Dr. Caſtellani of Mantua; 2. A hiſtory of an habitual colic cured by the uſe of coffee, by Dr. John Baptiſt Derinch; 3. An eſſay on the means of reſtoring drowned perſons, by a member of the academy at Mantua; 4. An account of a goat with two heads, by Dr. Dominico Caranio, profeſſor of phyſic at Cantiano; 5. An account of the Oleum Ricini, preſented to the Medico-botanical Society at Florence, by Dr. Matthew Mederer, Profeſſor of Phyſic and Surgery.

18. Hiſtoire de la Société Royale des Sciences etablie à Montpellier, avec les Memoires de Mathematique et de Phyſique, tirés des Regiſtres de cette Société. *i. e.* Hiſtory of the Royal Society of Sciences at Montpellier, with the Mathematical and Philoſophical Memoirs, taken from from the Regifters of that Society. Vol. II. 4to. Montpellier, 1781. 742 pages.

The firft volume of this work appeared in 1766. This fecond volume brings it down to the year 1775. The hiftorical part contains a great number of detached obfervations, the whole of which it was not thought right to publifh. There are likewife nine eulogies, and among them that of the late M. Chirac.

19. Uber die behandlung der Gonorrhee. *i. e.* On the Treatment of the Gonorrhœa. 8vo. Augfburg, 1781. 80 pages.

This anonymous writer throws no new light on the difeafe.

20. Geffundheits Katechifmus fur das landvolk. *i. e.* Catechifm of Health for the ufe of country people. By *A. Senfft*, M. D. and Prof. at Wurtzburg, 8vo. Berlin, 1781. 564 pages.

21. Della Condizione de medici preffo gli antichi. *i. e.* Of the condition of phyficians among the ancients. By *John Benvenuti*, M. D. 8vo. Peroufa, 1780.

The author endeavours to prove, that phyfic, fo far from having been left to flaves, as Dr. Middleton and others have contended, was always held in the higheft efteem by the Egyptians, Greeks, and Romans.

22. Saggio

22. Saggio dell' Istoria Erbaria degl' Alpi di Pistoja, Modena, e Lucca; con nuove osservazioni botaniche e mediche. *i. e.* Specimen of the Botanic history of the Alps of Pistoja, Modena, and Lucca, with new botanic and medical observations. By *P. D. Fulgenzio Vitman*, Regius Professor of Botany in the University of Pavia. 8vo. Pavia.

23. Von dem Drehen der Schafe und dem Blasenbandwurme im Gehirne derselben, als der ursache dieser Krankheit. *i. e.* Of the vertigo of sheep, and of the *Tænia hydatigena* in the brain of sheep as a cause of that disease. By *Nathaniel Godfrey Leske*. 8vo. Leipsic, with a copper plate.

In dissecting sheep that died of this disease our author constantly found one or more hydatids in different parts of the brain, to which adhered a species of worm similar to those which Tyson has named *Lumbrici hydropici*, and Pallas *Tæniæ hydatigenæ*, but differing from them in this, that in the tæniæ of the brain, which our author calls *Multicipites*, several bodies and heads of a worm are found adhering to one hydatid, whereas in the others each worm has a distinct vesicle.

24. Geschickte der Wissenschafften in der Mark Brandenburg, &c. *i. e.* History of the

Sciences

Sciences in the Marquisate of Brandenburg, particularly of Physic, from the most remote period to the end of the 16th century. By *J. C. W. Mochsen*, M. D. Physician to the King of Prussia. 8vo. Berlin, 1782.

25. Anmerkungen uber die viehsuchen in Oesterreuk. *i. e.* Remarks on the diseases of cattle in Austria. By *John Wolstein*, M. D. Professor of Veterinary Physic at Vienna, 1781.

26. Remarques sur les fievres en general, et en particulier sur celles de l'automne 1781 et 1782, &c. *i. e.* Remarks on fevers in general, and particularly on those of the autumns of 1781 and 1782, addressed in the form of a letter to M. Colombier, of the Faculty of Physic at Paris. By M. *Daignan*, first physician to the army in Brittany. 8vo. Paris, 1783. 62 pages.

27. Cerebri Nervorumque Historia corporis humani anatome repetita cum duabus tabulis. Auctore *Joanne Gottlobio Haasio*, Medicinæ Professore publico extraordinario. 8vo. Leipsic, 1781. 134 pages.

28. Dissertatio Inauguralis de Filicum fructicatione. Auctore *Joanne Antonio Lammersdorf*. 4to. Gottingæ, 1782.

